# NLRP3 inflammasome inhibition protects against intracranial aneurysm rupture and alters the phenotype of infiltrating macrophages

**DOI:** 10.3389/fstro.2023.1202137

**Published:** 2023-07-19

**Authors:** William S. Dodd, Devan Patel, Kartik Motwani, Brandon Lucke-Wold, Koji Hosaka, Brian L. Hoh

**Affiliations:** ^1^Department of Neurosurgery, College of Medicine, University of Florida, Gainesville, FL, United States; ^2^Department of Neurosurgery, Jacobs School of Medicine and Biomedical Sciences, University of Buffalo, Buffalo, NY, United States

**Keywords:** cerebral aneurysms, subarachnoid hemorrhage, hemorrhagic stroke, NLRP3 inflammasome, macrophages

## Abstract

**Background:**

Aneurysmal subarachnoid hemorrhage is a devastating cerebrovascular disease associated with high morbidity and mortality. Macrophage-mediated mural inflammation is a key pathogenic component contributing to aneurysm rupture.

**Objective:**

To investigate the effect of pharmacological inhibition of the NLRP3 inflammasome on aneurysm rupture.

**Methods:**

Cerebral aneurysms were induced in C57BL/6 mice with a combination of hypertension and an intracranial dose of elastase. Mice were treated with either 40 mg/kg of MCC950 or saline via intraperitoneal injections. Vascular tissue at the circle of Willis was harvested for analysis via immunofluorescent microscopy or qPCR.

**Results:**

NLRP3^+^ cells are more common in the aneurysm tissue compared to the normal cerebral vasculature. The mRNA expression of the downstream NLRP3 pathway components caspase-1, IL-1β, and GSDMD is also increased in the aneurysm tissue compared to healthy vessels. There was no difference in the aneurysm formation rate between MCC950- and vehicle-treated mice; however, MCC950 treatment significantly reduced aneurysm rupture rate. There was no difference in systemic blood pressure between both groups. MCC950 treatment also extended the symptom-free survival of mice after aneurysm induction. Mechanistically, NLRP3 inhibition decreased the phenotype polarization of infiltrating macrophages without affecting the total number of macrophages in the vessel wall.

**Conclusions:**

Our results indicate that the NLRP3 inflammasome contributes to aneurysm rupture and macrophage polarization within the vessel wall. The NLRP3 pathway is a promising therapeutic target for the development of therapeutics to prevent aneurysmal hemorrhagic stroke.

## 1. Introduction

Cerebral aneurysms affect 3–5% of the population worldwide, and aneurysm rupture is the leading cause of non-traumatic subarachnoid hemorrhage (SAH) (Wardlaw and White, 2000). SAH is associated with high rates of mortality and long-term morbidity (Hop et al., 1997; Wardlaw and White, 2000; Janjua and Mayer, 2003; Brown and Broderick, 2014), thus an intervention prior to aneurysm rupture is ideal. Unfortunately, current standards of care, endovascular coiling, or microsurgical clipping also present their own risks, including nosocomial aneurysm rupture (Etminan and Rinkel, 2016). Accordingly, the pathophysiology of aneurysm rupture has been a field of intense investigation to develop non-surgical therapeutics to prevent aneurysm rupture and to reduce the disease burden of hemorrhagic stroke.

One central theme of such research is the pathogenic role of macrophages in the progression and rupture of aneurysms (Shimizu et al., 2019). Cells that are CD68^+^, a marker for macrophages and monocytes, are found in both human and rodent aneurysm tissue, and their abundance correlates with pathogenic wall remodeling (Kataoka et al., 1999; Tulamo et al., 2010). Further, macrophage depletion (Kanematsu et al., 2011) and deletion of macrophage-inducing chemokines (Aoki et al., 2009a) have been shown to suppress aneurysmal change in animal models. Disruption of NF-κB signaling in macrophages also prevents the formation of aneurysms (Aoki et al., 2017), further solidifying the notion that macrophage-mediated inflammation is deleterious in aneurysmal cerebral vessels. More recent work from our laboratory and others has shown that pro-inflammatory, M1-type macrophages are highly associated with aneurysm progression, and inhibiting specific inflammatory pathways can decrease macrophage burden and alter macrophage polarization in favor of an M2, anti-inflammatory phenotype (Shao et al., 2017; Nowicki et al., 2018; Wajima et al., 2019). Taken together, these findings suggest anti-inflammatory therapeutics that affect macrophage phenotype and function are promising targets for further research.

The NLRP3 inflammasome is one such target that we aimed to evaluate for its role in aneurysm rupture. In 2015, Zhang et al. (2015) conducted an *ex vivo* study of human aneurysms and found that the expression of NLRP3 and other inflammasome components correlated with aneurysm progression. NLRP3 is an intracellular innate immune receptor that complexes with ASC/PYCARD and caspase-1 in response to various inflammatory stimuli to form the so-called “inflammasome” (Lamkanfi and Dixit, 2014; Zhou et al., 2018). Once activated, the inflammasome is responsible for the post-transcriptional maturation of IL-1β and IL-18 (Martinon et al., 2002) as well as the pyroptosis effector protein gasdermin D (Shi et al., 2015). Zhang et al. also reported that NLRP3 co-localized with the macrophage marker CD68 within the wall of unruptured and ruptured aneurysms. While these findings are only correlative, they suggest that the NLRP3 inflammasome could be a favorable target for aneurysm research for several reasons. First, as an innate immune receptor and mediator, the inflammasome is canonically regulated by damage-associated molecular patterns (DAMPs) and other stimuli that are characteristic of aneurysmal change (Hernandez-Cuellar et al., 2012; Usui et al., 2015; Hoseini et al., 2018; Song and Li, 2018). Second, NLRP3 inhibition has already been shown to protect against other vascular diseases (Marchetti et al., 2014; van der Heijden et al., 2017). Third, the inflammasome is the primary regulator of mature IL-1β production, and IL-1β has well-documented contributions to aneurysm progression (Moriwaki et al., 2006; Aoki et al., 2009b). Fourth, a highly specific small molecule inhibitor of NLRP3, MCC950, is pharmacodynamically profiled and commercially available (Coll et al., 2015). Finally, NLRP3-NF-κB interactions are well documented, supporting the idea that NLRP3 inhibition could suppress the pro-inflammatory M1-type phenotype polarization of macrophages observed in aneurysms (Maruyama et al., 2019; Zhang et al., 2019).

In this study, we tested the effect of NLRP3 inhibition via MCC950 treatment on a mouse model of cerebral aneurysm rupture. We then analyzed the aneurysm tissue for macrophage infiltration, phenotype polarization, and mRNA expression of NLRP3 pathway components. We hypothesized that NLRP3 inhibition via MCC950 would protect against aneurysm rupture, decrease the M1/M2 macrophage ratio, and reduce the IL-1β expression in the vessel walls.

## 2. Materials and methods

### 2.1. Animals

All animal experiments were performed in accordance with our Institutional Animal Care and Use Committee (IACUC) and Animal Research: Reporting of In Vivo Experiments (ARRIVE) guidelines. All animals were 12–15-week-old female C57BL/6 mice (Charles River Laboratories, Wilmington, MA) and were housed in pathogen-free housing with *ad libitum* access to food and water and under a 12-h light/dark cycle. All animals underwent twice daily monitoring from institutional animal care services staff and/or investigators. Mice were randomized to MCC950 or vehicle groups prior to the first surgery.

### 2.2. Murine cerebral aneurysm model

Cerebral aneurysms were induced as previously described (Hosaka et al., 2014; Hoh et al., 2018). Briefly, mice were anesthetized with ketamine (100 mg/kg) and xylazine (10 mg/kg) before the left common carotid artery and the right renal artery were ligated with 7-0 silk suture (Ethicon Inc., Somerville, NJ). Carotid and renal artery ligations are hereafter referred to as ligations surgery. One week after ligations surgery, mice were anesthetized and 10 μL of 1.0 U/mL porcine pancreatic elastase solution (Worthington Biochemical Corp, Lakewood, NJ) diluted in phosphate buffered saline (PBS, Invitrogen, Carlsbad, CA) was stereotactically injected into the right basal cistern at 1.2 mm rostral of bregma, 0.7 mm lateral of midline, and 5.3 mm deep to the surface of the brain. A subcutaneous osmotic pump (Model 2004, Alzet, Cupertino, CA) was placed in the right flank immediately after elastase injection and continually infused with angiotensin II (Bachem, Torrence, California) at a dose of 1,000 ng/kg/min for up to 3 weeks. Elastase injection and osmotic pump placement are hereafter referred to as aneurysm induction. Following recovery from aneurysm induction surgery, mice were fed a diet of 8% NaCl with 0.12% beta-aminopropionitrile (BAPN; Harlan Laboratories, Indianapolis, IN).

Three weeks post-aneurysm induction, mice were deeply anesthetized with ketamine/xylazine. A bilateral anterolateral thoracotomy with transverse sternotomy was made to expose the heart and great vessels. The right atrium was punctured with the tip of a 23-g needle as an outlet before the left ventricle was sequentially perfused with normal saline, 4% paraformaldehyde, and Coomassie Brilliant Blue dye in a 20% gelatin solution. Brains were carefully removed from the skull and inspected under a dissection microscope for the presence of aneurysms as evidenced by an outpouching of cerebral arteries in or connected to the circle of Willis. Aneurysm rupture was identified by the presence of extravasated blood near an identified aneurysm. Any mouse exhibiting stroke-like neurological symptoms (inactivity, circling paresis, or ≥15% weight loss) < 3 weeks post-aneurysm induction was immediately euthanized, and the brains were inspected for evidence of hemorrhage or aneurysm rupture. Mice that died suddenly prior to the 3 weeks post-aneurysm induction were inspected for evidence of intracranial hemorrhage and aneurysm rupture but were not included in histological studies. Mice were randomly allocated to either MCC950 or vehicle (saline) treatment groups, with a total of 25 mice in each group. The total mortality rate due to surgical procedures was 9/out of 50 (18%). Two mice (4%) were also excluded from the analysis due to significant tissue destruction prior to carcass discovery and examination that prevented the classification of the cerebrovascular tissues.

### 2.3. Pharmaceutics and dosing

MCC950 was obtained from Selleckchem (Catalog No.S7809, Houston, TX) and diluted in 0.9% NaCl in water. Then, 0.9% saline was used as the vehicle control for all experiments. MCC950 (40 mg/kg) or an equivalent volume of vehicle was administered daily via an intraperitoneal injection beginning 2 days before the elastase injection.

### 2.4. Blood pressure measurements

Arterial blood pressure was measured using the non-invasive CODA mouse tail-cuff blood pressure monitoring system (Kent Scientific, Torrington, CT). Mice were anesthetized via isoflurane inhalation for blood pressure recordings at days −1 (baseline), 5, 15, and 27 post-ligations. Mean arterial pressure (MAP) was calculated as 1/3^*^systolic blood pressure + 2/3^*^diastolic blood pressure. Each recorded MAP value was the average of three consecutive readings per animal.

### 2.5. Immunofluorescence microscopy

Following perfusion-fixation and overnight post-fixation in 4% PFA, brain samples were embedded in paraffin wax and cut into 7 μm sections with a Leica RM2125 RTS microtome (Leica Microsystems, Buffalo Grove, NY) and adhered to poly-L-lysine-coated glass slides. Serial sections were deparaffinized, rehydrated, and washed with 1x Tris-buffered saline with 0.05% Tween-20 (TBST) before being incubated overnight with one or more of the following antibodies: rabbit anti-NLRP3 (bs-10021R, Bioss Antibodies, Woburn, MA), rat anti-F4/80 (MCA497R, BioRad, Hercules, CA), rabbit anti-iNOS, and goat anti-Arginase I (ab15323 and ab60176 respectively, Abcam, Cambridge, MA). Primary antibodies were detected using donkey anti-rabbit IgG AlexaFluor594, donkey anti-rabbit IgG AlexaFluor488, donkey anti-rat IgG AlexaFluor594, or donkey anti-goat IgG AlexaFluor488 (Cat #s: A32754, A32790, A48271, and A32814, respectively, ThermoFisher, Waltham, MA). Slides were mounted with DAPI-containing mounting medium (VectaShield, Vector Labs, Burlingame, Ca.) and imaged using an Olympus IX71 fluorescent microscope (Olympus America, Center Valley, PA). All images were analyzed by two blinded observers. M1/M2 ratio was calculated as the average percentage of M1 macrophages divided by the average percentage of M2 macrophages in each sample.

### 2.6. mRNA reverse transcription and quantitative PCR

Ten mice were randomly assigned to either aneurysm induction or sham surgery (no exposure to elastase), and mRNA was extracted from the murine circle of Willis at 7 days post-aneurysm induction. Murine cerebrovascular tissue from the circle of Willis was carefully dissected away from the brain under a dissecting microscope before being flash-frozen in liquid nitrogen and homogenized using a Dounce homogenizer. The homogenized cerebrovascular tissue was then used for mRNA extraction using a binding column-based kit following the manufacturer‘s recommended protocol (Cat. #: 74104, Qiagen, Hilden, Germany). and reverse transcription was performed using a kit obtained from New England Biolabs (Cat. #: E6560, Ipswich, MA). cDNA was amplified using a master mix kit (Cat. #: 1725271, BioRad, Hercules, CA) and the following primers: *Hprt1* F: 555^′*′′*^-CTGGTTAAGCAGTACAGCCCCAA-333^′*′′*^, *Hprt1* R: 555^′*′′*^-CGAGAGGTCCTTTTCACCAGC-333^′*′′*^, *Nlrp3* F: 555^′*′′*^-TCTGCACCCGGACTGTAAAC-333^′*′′*^, *Nlrp3* R: 555^′*′′*^-CATTGTTGCCCAGGTTCAGC-333^′*′′*^, *Casp1* F: 555^′*′′*^-TGCCTGGTCTTGTGACTTGG-333^′*′′*^, *Casp1* R: 555^′*′′*^-GTCACCCTATCAGCAGTGGG-333^′*′′*^, *Il1b* F: 555^′*′′*^-CCCAAAAGATGAAGGGCTGC-333^′*′′*^, *Il1b* R: 555^′*′′*^-TGATGTGCTGCTGCGAGATT-333^′*′′*^, *Gsdmd* F: 555^′*′′*^-TGCGTGTGACTCAGAAGACC-333^′*′′*^, *Gsdmd* R: 555^′*′′*^-CAAACAGGTCATCCCCACGA-333^′*′′*^. Relative cDNA abundance was calculated using the 2^−Δ*ΔCT*^ method.

### 2.7. Statistical analysis

**Figure 1:**
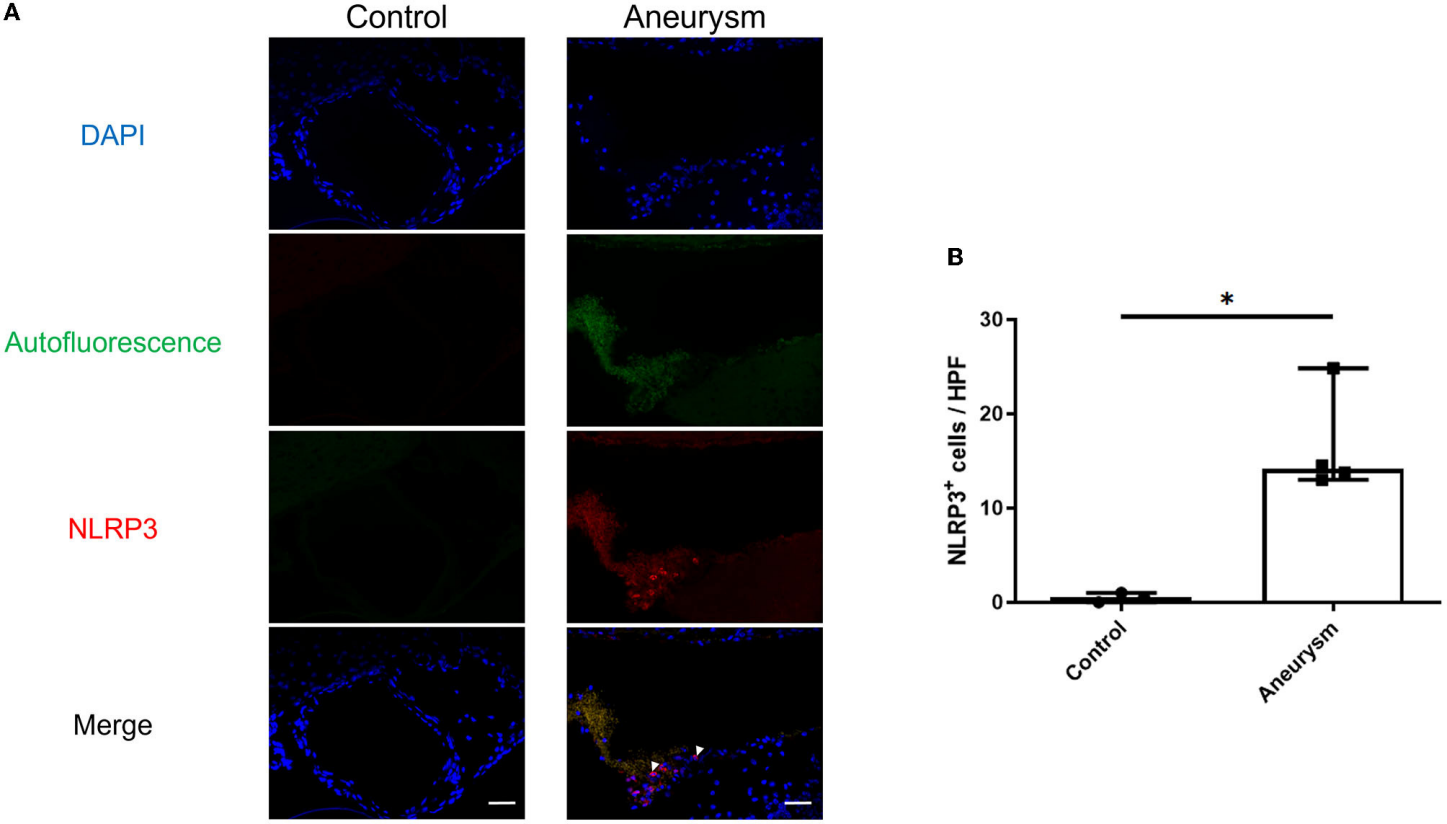
Aneurysms have increased NLRP3(+) cells at 1-week post-aneurysm induction compared to the normal cerebral vasculature. **(A)** Representative images of aneurysm **(right)** and normal vessel control **(left)** tissue probed for NLRP3. Arrowheads indicate NLRP3^+^ cells. Scale bar = 50 μm. **(B)** Image analysis of the number of NLRP3^+^ cells per high-powered field (HPF) with a box plot indicating median + 95% CI, *n* = 4 each, ^*^P < 0.05.

**Figure 2:**
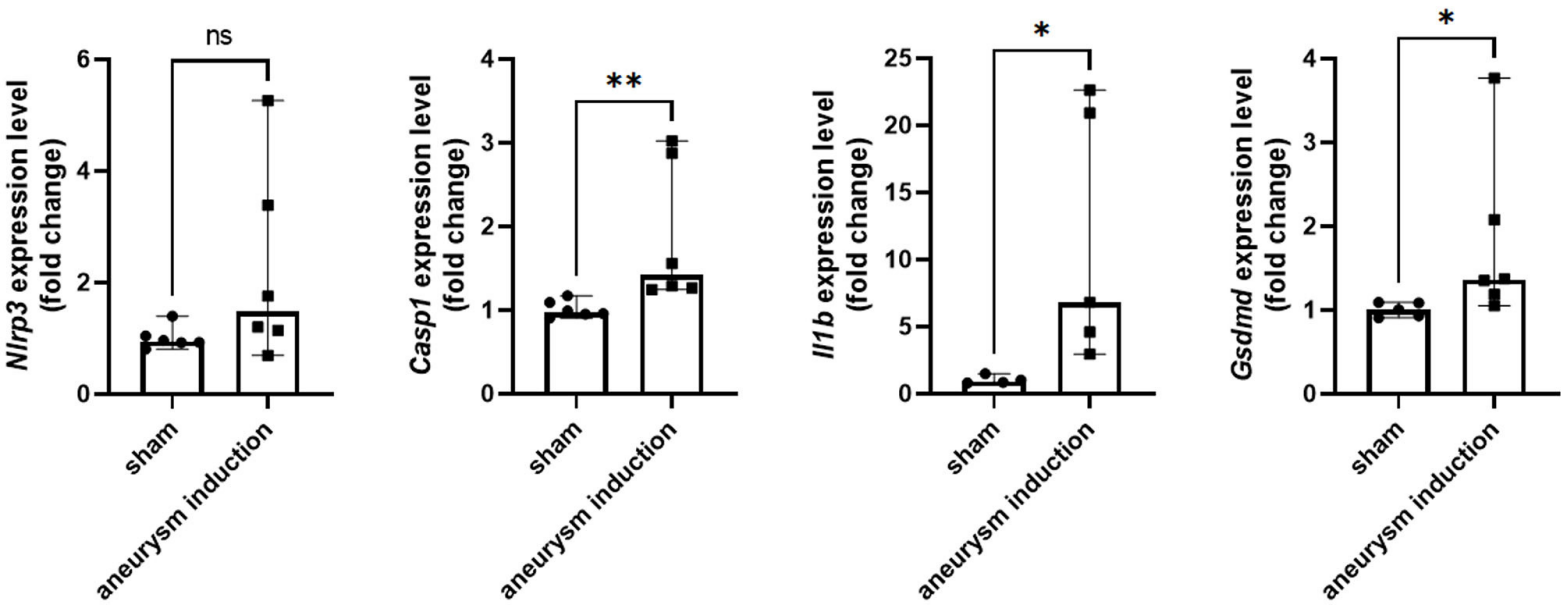
Aneurysm induction upregulates mRNA expression of inflammasome pathway components. *Nlrp3, Casp1, Il1b*, and *Gsdmd* were measured by RT-qPCR in the murine cerebrovascular tissue after sham operation or aneurysm induction. Expression levels shown as fold change relative to the sham group with box plots indicating median + 95% CI, *p < 0.05, **p < 0.01.

Statistical significance was considered to be a *p* < 0.05. All power calculations were completed with a β of 0.8 to detect a minimum biological difference of 30%. A comparison of aneurysm formation and rupture rates between groups were analyzed using Fisher's exact test. Survival data were analyzed using a Mantel–Cox log-rank test. Continuous variables compared between two groups (Figures 1, 2, **4**) were analyzed using the non-parametric Mann-Whitney *U* test and presented as median (95% confidence interval). All data analysis was performed using Prism data analysis software (GraphPad Software, San Diego, CA).

## 3. Results

### 3.1. The NLRP3 inflammasome is expressed in the cerebral aneurysm tissue

First, we investigated the expression of the NLRP3 pathway in aneurysm tissue vs. healthy cerebral vessels. Our aim for this experiment was to confirm NLRP3 expression in murine aneurysm tissue, as it has previously only been documented in human aneurysm tissue (Zhang et al., 2015). We analyzed vessels at seven days post-aneurysm induction, as this is when the rupture rate is highest based on our previous findings (Hosaka et al., 2014; Hoh et al., 2018; Nowicki et al., 2018). The aneurysm tissue had 14.1 (95% CI: 7.6–25.4) NLRP3^+^ cells per high-powered field compared to just 0.5 (95% CI: −0.7–1.7) cells/HPF in the healthy cerebral vessel control tissue (*p* = 0.01, *n* = 3–4 each) (Figure 1). On the mRNA level, aneurysm induction also stimulated the expression of inflammasome components Caspase-1 [sham: 1.0 (95% CI: 0.9–1.1) vs. aneurysm: 1.4 (95% CI: 1.0–2.8) relative expression units, p = 0.002, n = 5–6 each], IL-1β [sham: 0.9 (95% CI: 0.5–1.5) vs. aneurysm: 6.8 (95% CI: 0.1–23.3) relative expression units, *p* = 0.02, *n* = 5–6 each], and Gasdermin D [sham: 1.0 (95% CI: 0.9–1.1) vs. aneurysm: 1.4 (95% CI: 0.7–2.9) relative expression units, *p* = 0.02, *n* = 5–6 each] (Figure 2).

### 3.2. NLRP3 inflammasome inhibition reduces aneurysm rupture but does not affect aneurysm formation or systemic blood pressure

**Figure 3:**
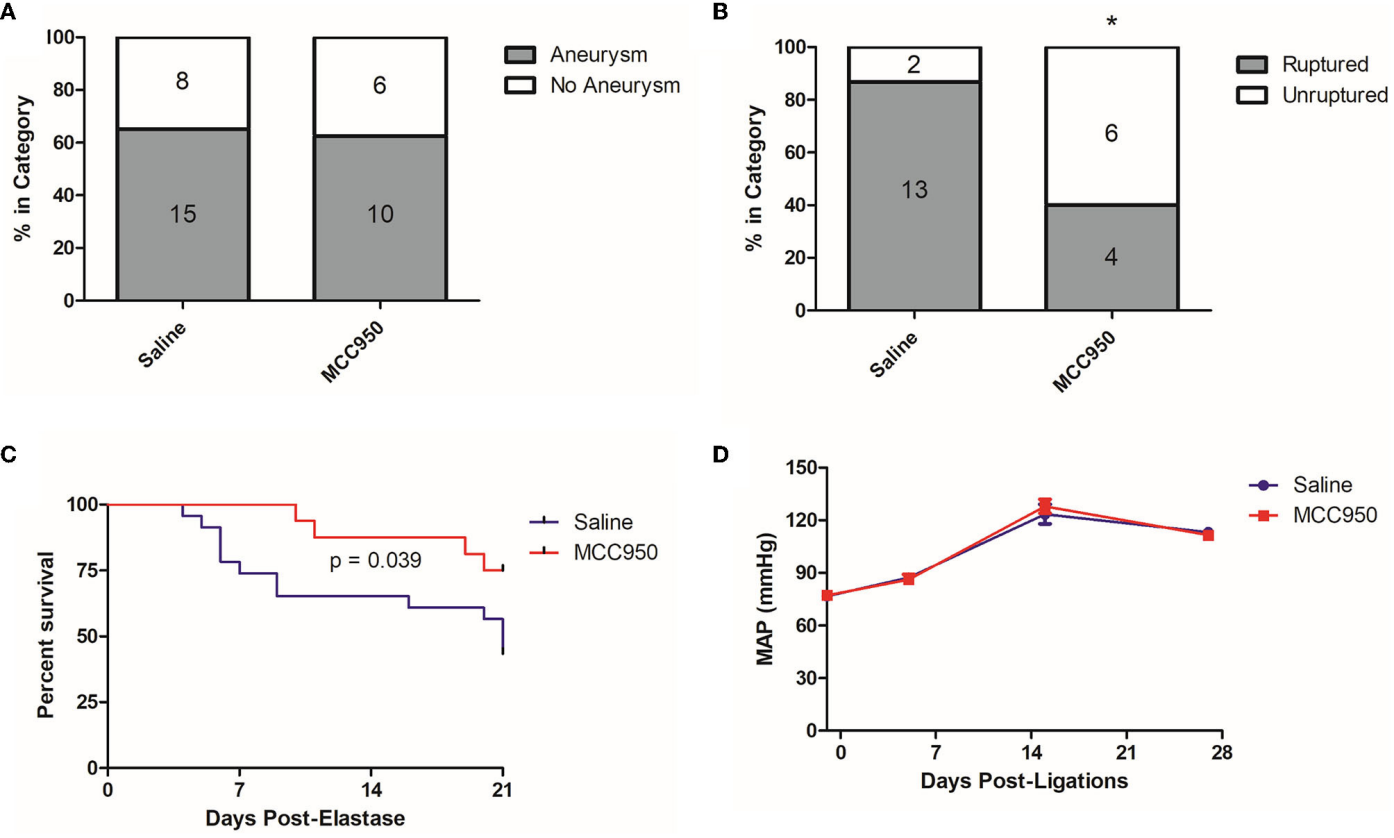
MCC950 treatment reduces the aneurysm rupture rate and extends symptom-free survival. **(A)** Aneurysm formation rates, **(B)** Aneurysm rupture rate, *p < 0.05 by Fisher's exact test. **(C)** Cumulative symptom-free survival, and **(D)** Mean arterial pressure, *p* = n.s. at all-time points.

To further study the relationship between NLRP3 and aneurysm rupture, we used our established murine model of cerebral aneurysm rupture. We allocated 10–12-week-old C57BL/6 mice to either MCC950 or saline treatment and induced aneurysms with a combination of hypertension and a single intracranial dose of elastase. MCC950 treatment did not affect aneurysm formation (65.2% in vehicle-treated group vs. 62.5% in MCC950-treated group, *p* = 1.00) (Figure 3A) but did significantly decrease the incidence of aneurysm rupture (86.7% in vehicle-treated group vs. 40.0% in MCC950-treated group, *p* = 0.04) (Figure 3B). NLRP3 inhibition also increased symptom-free survival compared to saline treatment (*p* = 0.039) (Figure 3C). Further, since hemodynamics and systemic blood pressure are central to aneurysm progression (Diagbouga et al., 2018), we wanted to rule out decreased systemic blood pressure as an indirect way that NLRP3 inhibition affects aneurysm rupture. We found that MCC950 treatment under our dosing regimen had no effect on mean arterial pressure before or after aneurysm induction. Mean arterial pressure in the vehicle-treated group was 76.6 ± 2.0 mmHg at baseline (1 day before ligations surgery), 87.3 ± 2.8 mmHg at 5 days post-ligations, 123.5 ± 5.6 mmHg at 15 days post-ligations, and 113.2 ± 2.7 mmHg at 27 days post-ligations vs. 77.1 ± 1.6, 86.2 ± 2.9, 127.9 ± 3.9, and 111.6 ± 1.7 mmHg at the same time points in the MCC950-treated group (*p* = 1.00 at all-time points, Figure 3D). These results showed that NLRP3 specifically contributes to aneurysm rupture through mechanisms other than decreased blood pressure.

### 3.3. NLRP3 inhibition alters the phenotype polarization of infiltrating macrophages after aneurysm induction

**Figure 4:**
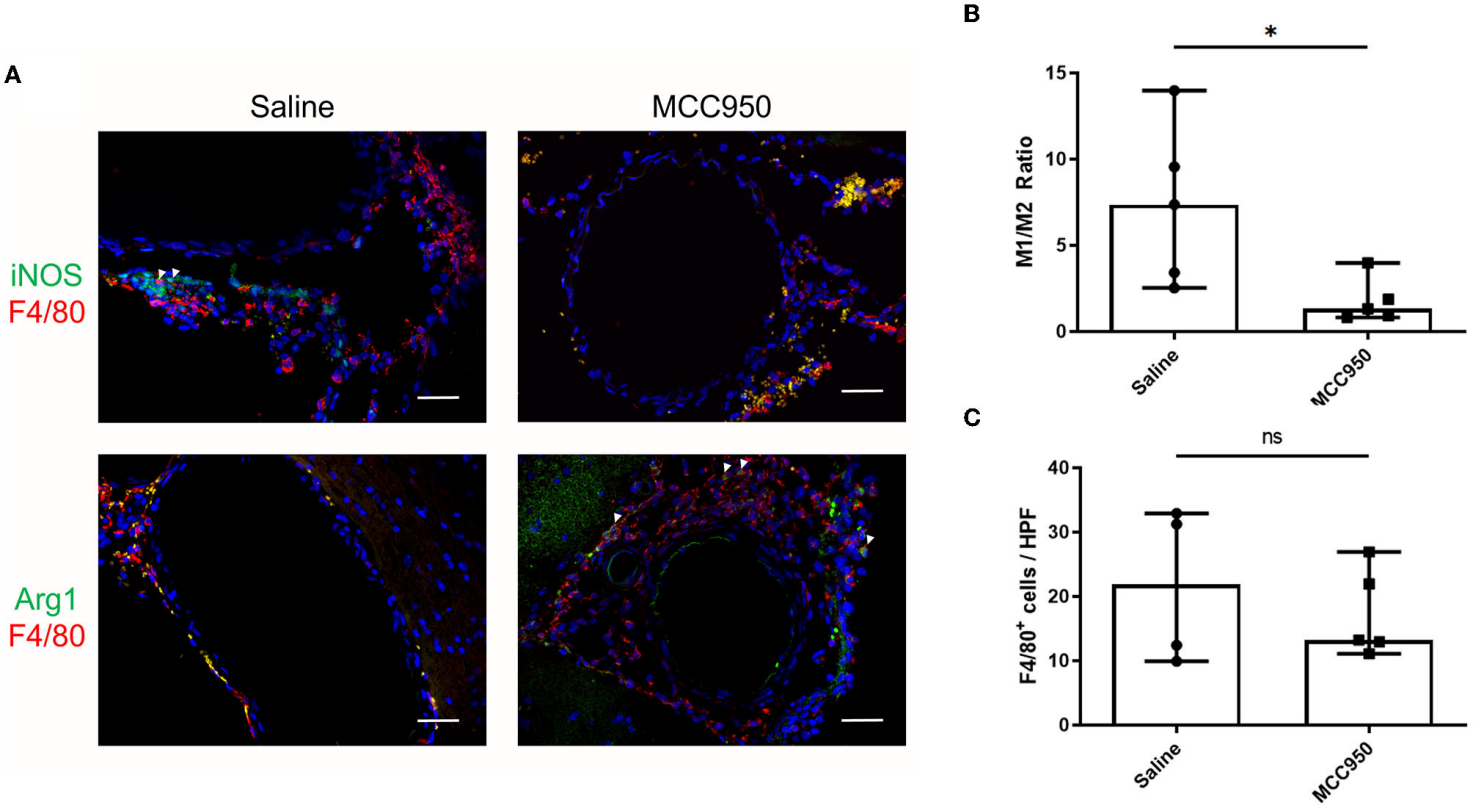
MCC950 treatment alters macrophage phenotype ratio in cerebral vasculature after aneurysm induction. **(A)** Representative images of M1-type **(top)** and M2-type **(bottom)** macrophages in saline-treated **(left)** and MCC950-treated **(right)** mice, scale bar = 50 μm. **(B)** Ratio of M1:M2 macrophage phenotypes in the vessel wall, *p < 0.05, *n* = 5 each. **(C)** Total number of F4/80^+^ cell per high-powered field, *n* = 5 each. All plots presented as median + 95% CI.

To elucidate the possible mechanisms by which MCC950 reduces aneurysm rupture, we next studied the cerebrovascular tissue of the mice collected at the end of the aneurysm induction protocol. We probed for M1- and M2-type macrophages using well-established markers, iNOS and Arg1, respectively (Wang et al., 2014). F4/80 was used to visualize all macrophages. Mice in the vehicle-treated control group had robust M1 macrophage infiltration (Figure 4A) compared to mice treated with MCC950, which had relatively more M2 macrophages (Figure 4A). Image analysis revealed that mice in the MCC950 group had a significantly lower ratio of M1- to M2-type macrophages in the aneurysm wall (1.4 (95% CI: 0.2–3.4) in MCC950-treated animals vs. 7.4 (95% CI: 1.6–13.2) in vehicle-treated animals, *p* = 0.03, *n* = 5 each) (Figure 4B). Next, we counted the total number of F4/80^+^ cells in the vessel wall of all samples to determine the effect of MCC950 treatment on the total macrophage burden. We found that there was no significant difference in macrophage infiltration between vehicle-treated and MCC950-treated samples [21.8 (95% CI: 2.3–40.9)] in vehicle-treated animals vs. 13.2 (95% CI: 8.7–25.8) in MCC950-treated animals, *p* = 0.51, *n* = 5 each) (Figure 4C).

## 4. Discussion

This study explored NLRP3 as a mediator of aneurysm pathology by testing the ability of the NLRP3 inhibitor MCC950 to prevent aneurysm rupture. We found that NLRP3^+^ cells are more abundant in murine aneurysm tissue than in the healthy cerebral vasculature (Figure 1), which supported the idea that NLRP3 was associated with aneurysm pathology. Further, aneurysm tissue also demonstrated higher mRNA expression levels of the NLRP3 pathway components caspase-1, IL-1β, and GSDMD (Figure 2). We hypothesized that NLRP3 inhibition via MCC950 treatment would impede aneurysm progression and rupture. Indeed, treatment with MCC950 reduced the rate of aneurysm rupture compared to vehicle control (Figure 3B). Analysis of the cerebral vessels of treated mice revealed that MCC950 altered the composition of macrophage phenotypes within the vessel wall in favor of a decreased M1/M2 ratio (Figure 4B). M1 polarization is consistent with inflammatory pathway activation (Wang et al., 2014) and has been shown to be associated with rupture in both human and murine aneurysms (Hasan et al., 2012; Nowicki et al., 2018). Thus, we considered a lower M1/M2 ratio to be favorable in the context of aneurysmal disease. As with all immunofluorescence microscopy experiments, the effect of the disease processes on the accumulation of autofluorescent materials (e.g., heme derivatives and lipofuscin) must be taken into account as well. Overall, these results support our hypothesis that NLRP3 inhibition prevents aneurysm rupture and suppresses pro-inflammatory macrophage phenotype polarization.

Our study has some technical limitations that confine the interpretation of our results. Conceptually, our data indicates that the pathology of aneurysm progression is associated with NLRP3 pathway regulation (Figure 2); however, we did not directly measure the effect of MCC950 treatment on this pathway. We used only female mice in these experiments, leaving open the possibility that MCC950 affects aneurysm rupture in a sex-dependent manner. Our results should also be considered in the context of statistical power limited by sample size and procedural limitations. The difference in the rupture rate between the MCC950- and vehicle-treated groups (Figure 3B) carries a fragility index of 1, suggesting that these experiments would benefit from replication, and further testing before our conclusions are considered beyond reasonable doubt. Further, although previous studies have demonstrated MCC950 to be a specific inhibitor of the NLRP3 inflammasome, we did not conduct any experiments to exclude off-target effects of MCC950 treatment. Future studies should utilize global and cell-specific knockouts of NLRP3 and other inflammasome components to clearly delineate the role of the inflammasome in aneurysmal disease.

It is well documented that circulating monocytes are attracted to the sites of vessel injury in response to signals from the endothelium and smooth muscle cells (Aoki et al., 2016; Shimizu et al., 2019). Once extravasated, the circulating monocytes differentiate into tissue macrophages, which can adopt a variety of phenotypes and contribute to inflammation and repair in different ways. Macrophage phenotypes are heterogeneous and dynamic but can be analyzed in the M1-M2 paradigm based on the nitrogen metabolism pathways (Wang et al., 2014). Pro-inflammatory M1 macrophages express higher levels of inducible nitric oxide synthase, shuttling arginine toward cytotoxic NO production, while M2 macrophages express higher levels of Arginase I, promoting ornithine production, decreased NO synthesis, and tissue repair. Our results were consistent with a conceptual model that monocyte/macrophage NLRP3 inhibition directly alters their activation in a manner that hinders M1 polarization. This hypothesis is strengthened by an *in vitro* study on macrophage cells demonstrating that compounds that inhibit macrophage NLRP3 directly alter the expression of canonical M1 and M2 markers in favor of a decreased M1/M2 ratio (Zhang et al., 2018).

It remains possible, however, that the pharmacologic inhibition of NLRP3 with MCC950 causes decreased aneurysm rupture by acting on cell types other than monocyte/macrophages. NLRP3 activation in endothelial cells and vascular smooth muscle cells (VSMCs) is becoming increasingly acknowledged as an important mediator of inflammatory disease pathology (Ren et al., 2018; Wu et al., 2018). Our observations that MCC950 treatment affects M1/M2 phenotype polarization could be an indirect effect that works through suppressing endothelial- and VSMC-mediated inflammation. However, our finding that the total number of macrophages infiltrated into the vessel wall is unaffected by MCC950 treatment supports the idea that NLRP3 inhibition does not change the recruitment of monocytes to the site of vessel injury (Figure 4C).

## Data availability statement

The original contributions presented in the study are included in the article/supplementary material, further inquiries can be directed to the corresponding author.

## Ethics statement

The animal study was reviewed and approved by University of Florida Institutional Animal Use and Care Committee.

## Author contributions

WD, DP, KH, and BH conceived and designed experiments. WD, DP, KM, BL-W, and KH collected data. WD, DP, KM, BL-W, KH, and BH performed data analysis. WD, KH, and BH interpreted the results. WD drafted the manuscript. All authors critically revised the manuscript and approved the final manuscript.
